# Case Report: Thrombotic-Thrombocytopenic Purpura Following Ipilimumab and Nivolumab Combination Immunotherapy for Metastatic Melanoma

**DOI:** 10.3389/fimmu.2022.871217

**Published:** 2022-04-20

**Authors:** W. J. Mullally, F. J. Cooke, I. M. Crosbie, S. Kumar, V. E. Abernethy, E. J. Jordan, M. O’Connor, A. M. Horgan, R. Landers, J. Naidoo, P. M. Calvert

**Affiliations:** ^1^ Department of Medical Oncology, University Hospital Waterford, Waterford, Ireland; ^2^ Department of Colorectal Surgery, University Hospital Waterford, Waterford, Ireland; ^3^ Department of Radiology, University Hospital Waterford, Waterford, Ireland; ^4^ Department of Hematology, University Hospital Waterford, Waterford, Ireland; ^5^ Department of Nephrology, University Hospital Waterford, Waterford, Ireland; ^6^ Department of Pathology, University Hospital Waterford, Waterford, Ireland; ^7^ Royal College of Surgeons in Ireland (RCSI), Department of Medical Oncology, Beaumont Hospital, Dublin, Ireland; ^8^ RCSI University of Health Sciences, Dublin, Ireland; ^9^ Department of Medical Oncology , Sidney Kimmel Comprehensive Cancer Center at Johns Hopkins University, Bloomberg-Kimmel Institute for Cancer Immunotherapy, Baltimore, MD, United States

**Keywords:** combination checkpoint inhibitors, ipilimumab, nivolumab, hematological adverse events, thrombotic-thrombocytopenic purpura

## Abstract

A man in his early 50s presented with small bowel obstruction, requiring emergency laparoscopic small bowel resection for the metastatic melanoma of the jejunum with no identifiable primary lesion. One week after his first treatment with ipilimumab and nivolumab, he presented with diffuse abdominal pain, constipation, and fatigue. A computerized tomography scan did not identify a cause for his symptoms. This was rapidly followed by thrombocytopenia on day 11 and then anemia. He commenced intravenous corticosteroids for a suspected diagnosis of immune-related thrombocytopenia. On day 15, a generalized onset motor seizure occurred, and despite plasmapheresis later that day, the patient died from fatal immune-related thrombotic thrombocytopenic purpura (TTP). This was confirmed with suppressed ADAMTS13 (<5%) testing on day 14. Immune-related TTP is a rare and, in this case, fatal immune- related adverse event. Further studies are required to identify additional immunosuppressive management for immune-related TTP.

## Introduction

The use of combination immune checkpoint inhibitors (ICIs) with ipilimumab (anticytotoxic T-lymphocyte–associated antigen 4 monoclonal antibody) and nivolumab (anti-programmed cell death ligand-1) in advanced melanoma has demonstrated a 6.5-year overall survival (OS) of 49% (1). Approximately one-third (32%) of patients in the pivotal CheckMate 067 trial harbored a *BRAF* mutation. The 6.5-year OS in this subgroup exceeded the study population median OS at 57% (1), demonstrating that ICI therapy is an effective treatment for BRAF-mutated melanoma. The approved dosing used in the pivotal CheckMate 067 trial consists of induction with intravenous (IV) ipilimumab 3 mg/kg and nivolumab 1 mg/kg every 3 weeks for four doses, followed by maintenance nivolumab 3 mg/kg every 2 weeks.

Combination immunotherapy has also revolutionized patient outcomes in metastatic renal cell carcinoma (RCC). A meta-analysis including six trials (2) using immunotherapy doublet or single-agent ICI in combination with angiogenic or multikinase inhibitors identified more than tripled complete response rate when compared with sunitinib and a 26% decreased risk of death. This strategy has also demonstrated success in metastatic hepatocellular carcinoma (HCC), where its hostile immunosuppression tumor microenvironment led to no successful improvements to sorafenib for over a decade. The IMbrave 150 trial shows an 18-month OS of 52% with atezolizumab and bevacizumab and 40% with sorafenib (3). Similarly, there is an early promise of clinically meaningful benefit with the pembrolizumab and lenvatinib combination (4). However, these treatment strategies are associated with unique toxicities. The meta-analysis (5) including four metastatic RCC trials of combination immunotherapy identified over three times higher risk of all-grade pruritus and rash, although with 51%, 78%, and 40% reduction in grade 3–4 fatigue, all-grade palmar-plantar erythrodysesthesia, and all-grade nausea.

There were 59% grade 3+ immune-related adverse events (irAEs), 31% of which led to treatment discontinuation in the CheckMate 067 trial. The most frequently reported grade 3+ irAEs that occur from ICI include hepatic, gastrointestinal, endocrine, and dermatological events (6%–20%). The incidence of thrombotic-thrombocytopenic purpura (TTP) in patients with melanoma treated with ICIs is estimated at less than 1%, based on real-world data (6).

We present the case of a 51-year-old man who developed immune-related TTP following treatment with one dose of combination ipilimumab and nivolumab.

## Case Description

**Figure 1:**
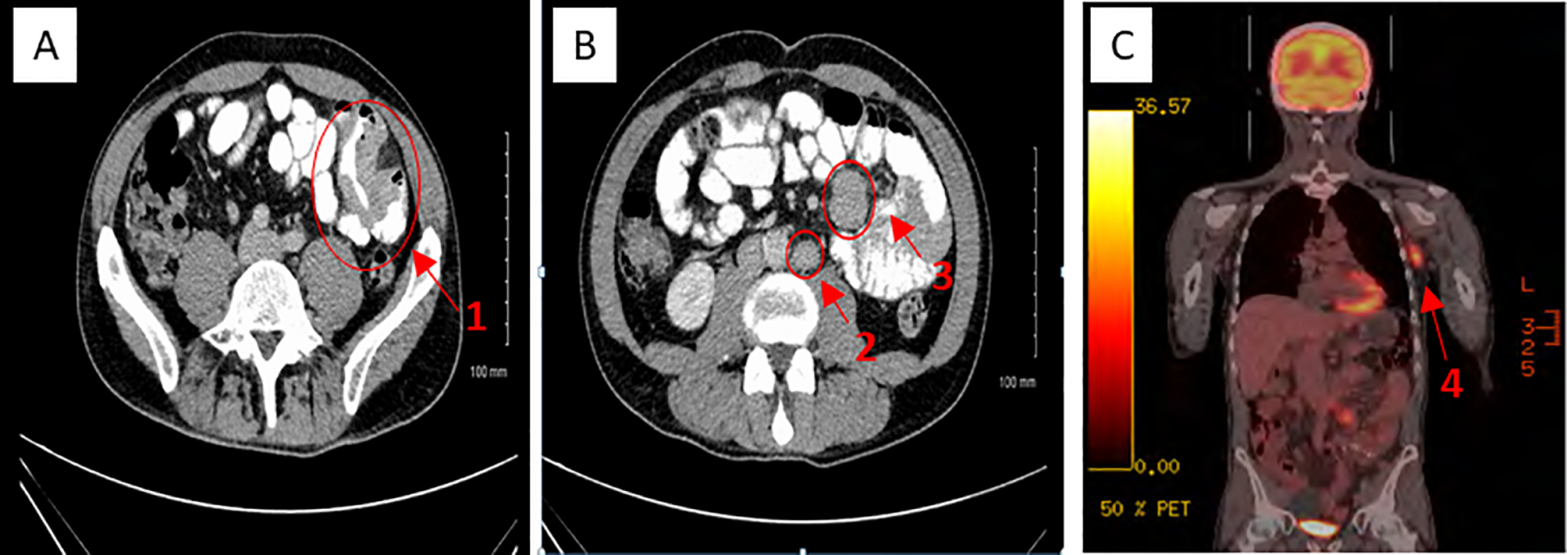
CT **(A, B)** and PET-CT **(C)** appearances of metastatic jejunal melanoma. 1 = Metatstatic jejunal small bowel obstruction. 2 = Retroperitoneal lymphadenopathy. 3 = Multifocal small bowel mesenteric melamona. 4 = Metastatic left axillary lymphadenopathy.

A man in his early 50s presented with a 2-month history of left iliac fossa pain, weight loss (14 kg), and mild fatigue. His past medical history was relevant for moderate gastritis and hiatus hernia (diagnosed on an esophagogastroduodenoscopy). His biochemical profile was unremarkable, apart from a mildly elevated C-reactive protein (16 mg/l). A contrast-enhanced computerized tomography (CT) scan (**Figure 1**) identified a thickened small bowel wall with mesenteric and retroperitoneal lymphadenopathy. A laparoscopic small bowel resection was completed, and a diagnosis of metastatic *BRAF V600E* mutant melanoma involving the jejunum was confirmed.

The patient’s postoperative recovery was complicated by an anastomotic leak and mesenteric hematoma requiring emergency relaparotomy and further small bowel resection. He was discharged 19 days after the initial surgery and referred to Medical Oncology for the consideration of systemic therapy.

Full skin examination and baseline staging positron emission tomography–computed tomography (PET-CT) showed no evidence of primary cutaneous melanoma (**Figure 1**) or other measurable diseases apart from left axillary lymphadenopathy (3.6 cm), small bowel mesenteric and retroperitoneal lymphadenopathy. As he had undergone extensive small bowel resection, we were concerned about his ability to tolerate oral BRAF and MEK inhibitor targeted therapy and therefore recommended first-line immunotherapy.

**Figure 2:**
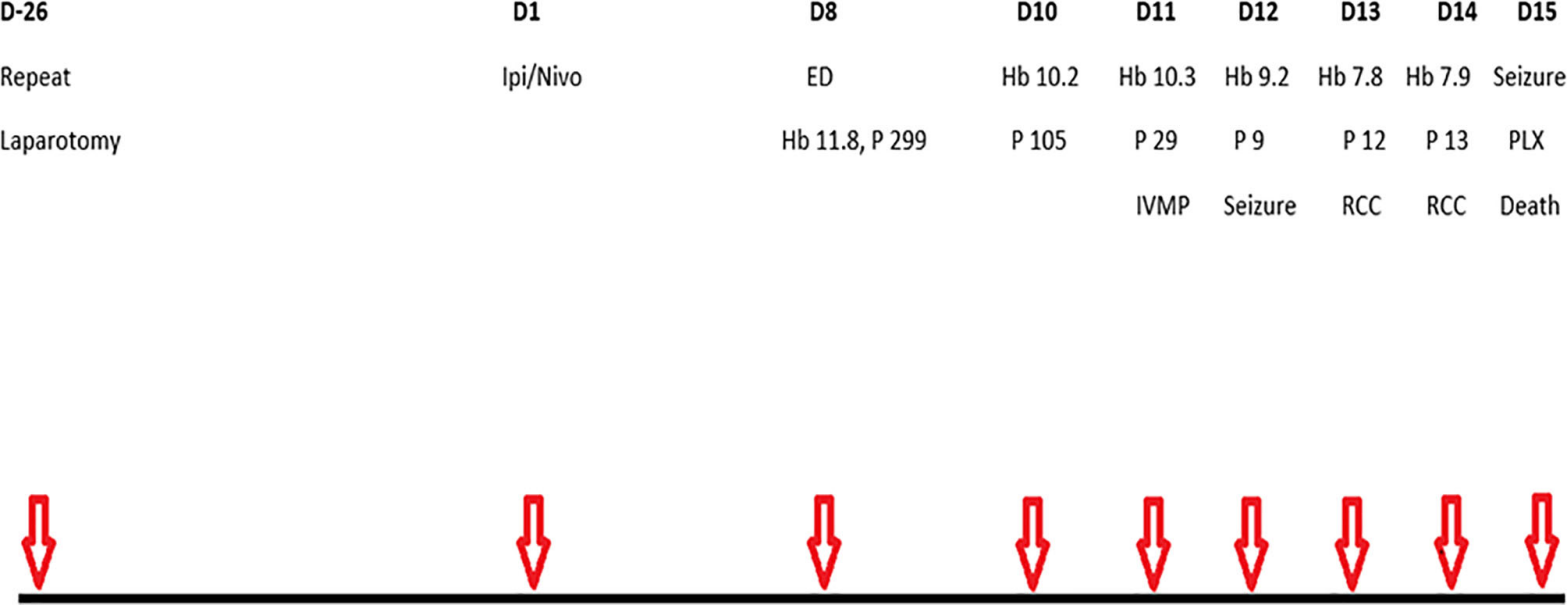
Schematic of main events. D, day; Ipi, ipilimumab; Nivo, nivolumab; ED, emergency department presentation with abdominal pain and fatigue; Hb, haemoglobin (g/dl); P, platelets (X10^9^/l); IVMP, intravenous methylprednisolone; RCC, red cell concentrate, PLX, plasmapheresis.

He commenced his first cycle of ipilimumab and nivolumab, 26 days after repeat laparotomy. Serial laboratory test results are annotated in **Table 1**. One week later (day 8), he represented with severe abdominal pain, constipation, grade 1 anorexia, and grade 2 fatigue (**Figure 2**). His C-reactive protein was 74 mg/L, and he was treated with IV fluids and broad-spectrum IV antimicrobials. The urgent contrast-enhanced CT scan of abdomen and pelvis revealed previously known small bowel mesenteric lymphadenopathy and retroperitoneal lymphadenopathy. Two days after hospitalization (day 10), the patient’s platelet count and hemoglobin level began to decline (**Table 1**). Following a hematology consultation on day 11, he commenced taking 1 mg/kg (50mg) of IV methyl prednisolone (MP) for a suspected immune-related thrombocytopenia (platelets 29 × 10^9^/L) as per ASCO guidelines (7). On day 12, he was found to have a worsening thrombocytopenia (9 × 10^9^/L), grade 2 anemia (9.2 g/dl), elevated lactate dehydrogenase (680 U/L), reduced haptoglobin (<0.10 g/L), and elevated schistocytes (4%) on the blood film consistent with a diagnosis of microangiopathic hemolytic anemia (MAHA). In addition, the patient developed new acute kidney injury (creatinine increased from 69 to 134 µmol/L), which was managed with IV fluids (0.9% sodium chloride).

**Table 1:** Patient case – daily laboratory parameters consistent with evolving TTP following cycle 1 of ipilimumab and nivolumab.

	Reference Range	Day 1	Day 8	Day 9	Day 10	Day 11	Day 12	Day 13	Day 14	Day 15		
										06:00	10:00	22.30
Hemoglobin	13 – 17 g/dl	11.1	11.8	11.7	10.2	10.3	9.2	7.8	7.9	7.8	8.3	7.6
White Blood Cell Count	4 – 10 X 10^9^/l	6.6	9.7	7.6	7.9	8.0	7.3	6.2	8.8	9.0	10.2	14.7
Neutrophils	2-7 X 10^9^/l	3.40	6.68	4.89	5.17	4.59	3.95	4.02	5.41	5.68	7.89	11.40
Lymphocytes	1-3 7 X 10^9^/l	2.09	1.65	1.41	1.60	2.27	2.05	1.57	2.60	2.32	1.48	1.48
Reticulocytes	50 – 100 X 10^9^/l	–	–	–	–	–	14.7	14.3	–	–	69.3	80.8
Platelets	150-400 X 10^9^/l	481	299	326	105	29	9	12	13	11	8	8
Prothrombin time	8.7-12.7s	–	11.0	10.6	–	11.3	–	–	10.8	11.1	11.1	11.8
Creatinine	62-106 µmol/l	59	53	59	57	69	134	135	122	104	114	130
Alanine Aminotransferase	5-41 U/l	16	10	9	15	16	14	15	18	15	15	
Total Bilirubin	2-21 µmol/l	2.7	7.4	5.6	17.2	30.2	25.2	28.9	34.6	28.4	41.4	
Albumin	35-50 g/l	42	42	41	33	32	30	29	33	30	34	34
Thyroid-stimulating hormone	0.27 – 4.2 mIU/l	1.5					2.6					
Lactate dehydrogenase	10-250 U/l	182					680	655			958	
Haptoglobin	0.3 – 2.0 g/l						<0.10	<0.10				
C-reactive protein	0-5 mg/l		73.9	75.5	69.6	71.5	53.6	33.4	19.2	9.4	9.9	7.8
Schistocytes	%						4	5.7			10.7	
ADAMTS-13	%								< 5.0			

Day 1 is the first day of Cycle 1 Ipilimumab and Nivolumab.

Later that day, he experienced a transient episode of confusion, and a brain CT identified a subacute infarction. He was unsuitable for cerebral arterial thrombolysis in the presence of thrombocytopenia. His condition deteriorated rapidly, requiring multidisciplinary input from Hematology, Nephrology, and Intensive Care. A petechial rash was evident on his lower limbs on day 13. He received one unit of packed red cells on days 13 and 14 for anemia with a hemoglobin level of 7.8 g/dl. Plasma testing for ADAMTS13 (A disintegrin and metalloproteinase with a thrombospondin type 1 motif, member 13) levels was performed on day 14. On day 15, he had a generalized onset motor seizure, and the subsequent brain CT showed no intracerebral hemorrhage. He received 1 g of Intravenous methylprednisolone (IVMP) prior to plasmapheresis. He developed another generalized onset motor seizure leading to cardiorespiratory arrest near the first plasmapheresis procedure and, despite full cardiopulmonary resuscitation, sadly died. The ADAMTS13 level was less than 5%, consistent with a diagnosis of TTP.

## Results

A subsequent autopsy identified microscopic thrombotic microangiopathy affecting small blood vessels in his lungs, epicardium, liver, and kidneys in keeping with a diagnosis of TTP, with no evidence of residual metastatic melanoma.

## Discussion

This case represents the first documented death from immune-related TTP following ipilimumab and nivolumab combination ICI therapy. The most consistent TTP signs of thrombocytopenia, MAHA, and seizure activity (8) were demonstrated. The suppressed ADAMTS13 level is pathognomonic. A deficient activity of ADAMTS13 leads to the release of uncleaved ultra large von Willebrand Factor (vWF) multimers into blood circulation. Ultralarge vWF multimers bind to platelets to form aggregates and subendothelial collagen exposed by endothelial damage yielding a pathologic meshwork of platelet-rich thrombi in the microcirculation (8), leading to end-organ microvascular injury, which accords with the autopsy findings. Suspected cases of this rare and potentially fatal irAE require early diagnosis and urgent plasmapheresis for favorable patient outcomes.

Thrombotic microangiopathies (TMAs) are heterogenous disorders characterized by disseminated thrombus formation in arterioles and capillaries, resulting in thrombocytopenia, MAHA, and potential end organ injury (8). The two TMA archetypes are TTP and hemolytic uremic syndrome (HUS) (9). In the absence of significant renal impairment, infection, hypertension, a history of autoimmune disease, and negative virology screen, HUS was readily excluded. It is imperative to differentiate MAHA from TTP-associated MAHA, as the former does not respond to plasmapheresis. Therefore, prompt exclusion of all other MAHA etiologies, including cancer-related MAHA, disseminated intravascular coagulation (DIC), and chemotherapy-induced MAHA, is essential. There was no clinical evidence of DIC in our patient, and in DIC, the platelet count rarely falls below 20 × 10^9^/L (9), unlike our patient. Cancer-related MAHA is rare (0.25–0.45 cases per million) (10) and associated with modestly reduced plasma ADAMTS13 activity (35%–84%) (11), which contrasts with our case of suppressed ADAMTS13 activity (<5%).

There is a lack of published data on the incidence and management of TTP and hematological irAEs in general from ICIs. The incidence of TTP in patients with melanoma treated with ICIs is estimated at less than 1% (n=11/2,360) from a US multi-institution retrospective study (6). This included patients with both early-stage and advanced melanoma receiving single-agent (n=7, 64%) or combination ICI (n=4, 36%).

The average time to onset of ICI-induced thrombocytopenia was 70 days (range 12–173 days), and the platelet count ranged from less than 5 to 104 × 10^9^/L. There were four patients who required immunosuppression (steroids and/or anti-CD20 therapy); three (75%) received ipilimumab (single agent or in combination), and one patient with anti-PD-L1 ICI. Unlike our case, the ADAMTS13 level was not recorded. Combination immunotherapy has also resulted in a case of TTP in a (12) metastatic renal cell carcinoma patient, which occurred early after ICI (day 9 of first treatment) therapy. Unlike our patient, she had received the CheckMate 214 advanced renal cancer dosing schedule of ipilimumab 1 mg/kg and nivolumab 3 mg/kg and her TTP responded to therapeutic plasma exchange, IVMP, and four doses of rituximab. The CheckMate 214 ICI regimen (13) was better tolerated with fewer grade 3+ irAE (46%) and treatment discontinuation (22%) when compared with the melanoma regimen.

The limitations of our case include no testing for the presence of ADAMTS13 autoantibody inhibitor, which would have further validated the TTP diagnosis, despite the confirmation of its suppressed activity (<5%). The option of proceeding with plasmapheresis earlier (day 11) may have potentially altered our patient’s outcome; however, there were many completing differentials including TTP. Other treatments including anti-CD20 or the more recently approved caplacizumab, an anti-vWF, licensed for acquired TTP could have been considered had our patient survived plasmapheresis. The pivotal HERCULES study (14) identified one death from cerebral ischemia that may have excluded the potential use of caplacizumab, owing to our patients’ cerebral infarct on day 12.

As our use of highly effective combination ICI increases across a wide range of cancer types, we are highlighting the risk of ICI-related TTP and importance of rapid diagnosis and management of this potentially fatal complication. Further evaluation of the efficacy of the ipilimumab 1 mg/kg plus nivolumab 3 mg/kg dosing schedule may be warranted in patients with advanced melanoma given the lower incidence of grade 3+ adverse events with this regimen. The exact mechanism and its rapid onset have yet to be elucidated. However, as our understanding of the complex interactions within the tumor microenvironment evolves, a potential biomarker identifying high-risk patients may be revealed. This will continue to be of more importance in the future as the incidence of TTP is expected to increase with ICI now used as adjuvant treatment (15–17) for many cancers.

## Author Contributions

WM: manuscript preparation. FC: consultant surgeon. IC: consultant radiologist. SK: consultant hematologist. VA: consultant nephrologist. RL: consultant pathologist. EJ, MO’C, AH, JN, and PC: review of manuscript. All authors contributed to the article and approved the submitted version.

## Conflict of Interest

JN: Research funding: AstraZeneca, Merck; Consulting/Advisory Board: AstraZeneca, Bristol Myers Squibb, Daiichi Sankyo, Kaleido Biosciences, Merck, NGM Pharmaceuticals, Pfizer, Roche/Genentech, Takeda. AH: Travel and conference grant from Amgen, Bayer, Pfizer, Janssen, Roche, Servier. WM: Travel and conference grant from Amgen, Eli Lilly, Janssen, Novartis, Pfizer, Roche, Servier. PC: Travel and conference grant from Bayer, Bristol Myers Squibb, Merck, Novartis, and Roche.

## Publisher’s Note

All claims expressed in this article are solely those of the authors and do not necessarily represent those of their affiliated organizations, or those of the publisher, the editors and the reviewers. Any product that may be evaluated in this article, or claim that may be made by its manufacturer, is not guaranteed or endorsed by the publisher.
